# The Visible Cement Data Set

**DOI:** 10.6028/jres.107.013

**Published:** 2002-04-01

**Authors:** Dale P. Bentz, Symoane Mizell, Steve Satterfield, Judith Devaney, William George, Peter Ketcham, James Graham, James Porterfield, Daniel Quenard, Franck Vallee, Hebert Sallee, Elodie Boller, Jose Baruchel

**Affiliations:** National Institute of Standards and Technology, Gaithersburg, MD 20899-0001, USA; Centre Scientifique et Technique du Batiment, Grenoble and Marne-la-Vallee, France; European Synchrotron Radiation, Facility, Grenoble, France

**Keywords:** cement hydration, image analysis, microstructure, Plaster of Paris, visualization, x-ray microtomography

## Abstract

With advances in x-ray microtomography, it is now possible to obtain three-dimensional representations of a material’s microstructure with a voxel size of less than one micrometer. The Visible Cement Data Set represents a collection of 3-D data sets obtained using the European Synchrotron Radiation Facility in Grenoble, France in September 2000. Most of the images obtained are for hydrating portland cement pastes, with a few data sets representing hydrating Plaster of Paris and a common building brick. All of these data sets are being made available on the Visible Cement Data Set website at http://visiblecement.nist.gov. The website includes the raw 3-D datafiles, a description of the material imaged for each data set, example two-dimensional images and visualizations for each data set, and a collection of C language computer programs that will be of use in processing and analyzing the 3-D microstructural images. This paper provides the details of the experiments performed at the ESRF, the analysis procedures utilized in obtaining the data set files, and a few representative example images for each of the three materials investigated.

## 1. Introduction

To produce materials with acceptable or improved properties, adequate characterization of their microstructure is critical. While the microstructure can be viewed in two dimensions at a variety of resolutions (e.g., optical microscopy, scanning electron microscopy, and transmission electron microscopy), it is usually the three-dimensional aspects of the microstructure that have the largest influence on material performance. Direct viewing of the three-dimensional microstructure is a difficult task for most materials. Confocal microscopy and atomic force microscopy can each provide three-dimensional information on surface topography, but not a complete representation of the bulk three-dimensional microstructure. For this task, the most relevant technique is that of three-dimensional (micro)-tomography.

Three-dimensional microtomography has been actively applied to the characterization of materials for about 15 years [1], with much interest by the oil industry in the characterization of porous rocks [2, 3]. More recently, it has been applied to building materials with applications to cement-based mortars [4, 5], building bricks [6], and concrete aggregates [7]. With microtomography, a complete three-dimensional image of the microstructure is obtained. As capabilities of the tomography systems have increased, it has now become possible to obtain such images with a resolution better than one micrometer per voxel (image element), at facilities such as the European Synchrotron Radiation Facility (ESRF) in Grenoble, France [8]. This resolution is of particular interest for cement-based materials, where the starting particles are typically micrometers and tens of micrometers in diameter and many hydration products are also formed at the scale of micrometers [9]. Imaging cement-based materials at a pixel size of 1 µm will allow direct comparison to current digital-image-based microstructural models which also operate at this scale [10, 11]. A variety of computational tools exist to directly calculate the transport and elastic properties of a three-dimensional image-based microstructure, when the corresponding properties of the component phases are known [12].

## 2. Experimental Procedure

For the experiments at the ESRF, Cement and Concrete Reference Laboratory (CCRL) cement 133, issued in June 1999, was used. This cement was selected because it has been well characterized in the CCRL proficiency sample testing program [13] and also via computer modelling [11]. Detailed information on the particle size distribution and mineralogical phase distribution for this cement can be found in the NIST Cement Images online database [14].

**Fig. 1:**
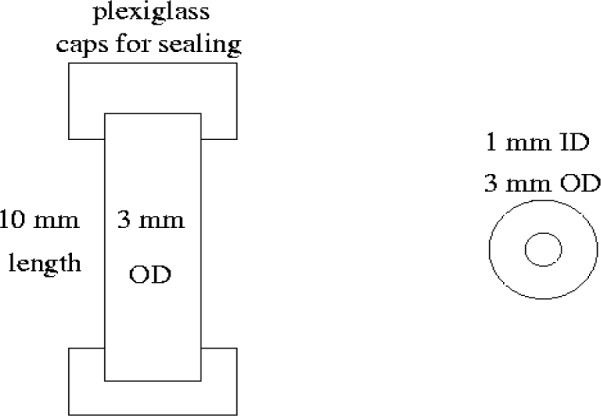
Schematic of plexiglass tubes for holding cement paste specimens.

To prepare a sample cement paste for viewing in the microtomography unit at the ESRF, the appropriate masses of cement (typically 50 g) and water were added to a small plastic beaker. The pastes were first mixed by hand for 1 min in the beaker, followed by 1 min of mixing using a “drill” mixer (a mixing blade attached to a regular electric drill). The sides of the beaker were then scraped and one final minute of drill mixing employed. Small pats of the paste were then carefully “extruded” into the sample tube molds, shown in Fig. 1. The molds were either capped or left open depending on the desired curing conditions (sealed, open to drying, or open with water periodically added on top to maintain saturation). In general, pastes with water-to-cement mass ratios (*w/c*) of 0.3 to 0.45 were prepared and viewed after various periods of hydration. The specific gravity of cement is typically about 3.2, so that a 50:50 volumetric proportion of cement and water would correspond to a *w/c* of about 0.31. In addition, samples of a packed dry cement powder were prepared and viewed using the microtomography unit. In some cases, after viewing the original dry packed powder, water was added to the sample tube mold and the hydrated “packed” cement paste was viewed after various hydration times. For most pastes, it was not possible to obtain a stable microtomography image prior to the setting of the cement paste (at about 4 h), due to local motion of cement particles within the pore solution. The only exception to this was the sample of cement paste that was first prepared as a dry packed powder in the tube mold, with the subsequent addition of water. For this specimen, we were able to obtain a reasonable image immediately after addition of the water, as the particles basically remained in their original packed configuration.

For the Plaster of Paris, a commercial locally-available product was utilized and the paste was prepared at a water-to-solids mass ratio (*w/s*) of 1.0. The low viscosity of these pastes allowed the tube molds to be easily filled. Plaster of Paris hydration was viewed after 4 h, 7 h, 15.5 h, and 5 d. In addition, a sample of the dry Plaster of Paris powder was placed in a separate tube mold and viewed using the microtomography setup.

The clinker brick was a hard-burned clay brick that had been examined previously using the ESRF microtomography unit [6] at a resolution of 6.67 µm per pixel. For this experiment, a small 2mm×2mm×2mm cube of the brick was carefully sawn from a larger specimen and mounted for viewing with the microtomography unit.

All samples were imaged on the 3-D microtomography (µCT) unit developed on beamline ID 19 at the ESRF. The system utilizes a large monochromatic parallel beam and a 2-D area detector. The specimen to be imaged is mounted on a translation/rotation stage allowing precise alignment in the beam. To compile a 3-D image set for a material, a series of over 1000 radiographic images are recorded at different angular positions from 0° to 180°. After conversion to light by a flourescent scintillator screen, the radiographic images are digitized using a Frelon camera [15], which consists of a 2-D charge coupled device (CCD) array with 1024 × 1024 elements, covering an area of 19.5 mm by 19.5 mm. The distance from the sample to the scintillator is 8 mm. The Frelon camera has a resolution of 2 µm full width-half maximum. A 3-D filtered back-projection algorithm is then used to reconstruct a 3-D image of the specimen from the series of 2-D projections [16]. For the operating conditions and optical setup employed in this study, it was possible to acquire a 1024 × 1024 × 1024 image with a voxel dimension of 0.95 µm in just over 20 minutes (1.2 s per radiographic image). Including sample preparation, adjustment, and equipment setup, each data set required about 1 h of total clock time.

The (greylevel) intensity at each voxel in the final 3-D image corresponds to the linear attenuation coefficient of the material contained in that voxel. Thus, cement particles will appear much brighter than hydration products, which will appear brighter than water-filled or air-filled porosity.

## 3. Results

### 3.1 Data Content

The Visible Cement Data Set is organized into distinct subsets for each experiment performed at the ESRF in September 2000. Each distinct data set is comprised of a set of files that provide the raw data, a text-based description, 2-D and 3-D images of the microstructure, and movie files containing animated views of the microstructure. Details on each of these are as follows:

Raw data files—each raw datafile contains a 512 × 1024 × 1024 volume of data comprising either the top or bottom half of the complete microstructure. These files have been rescaled with a data resolution of one byte per data element, each element thus having a value (greylevel) between 0 and 255. The initial data provided by the ESRF was at a data resolution of four bytes (floating-point) per data element, with a proportionately higher greylevel resolution. All initial indications are that the reduced 0–255 greylevel range is adequate for reliably distinguishing features (phases) in the data sets. In both the raw data files available on the web site and the initial data files supplied by the ESRF, the right-most index varies the fastest in the file. That is the first 1024 data elements in the file correspond to the first row (left to right) of 3-D data and the first 1024 × 1024 data elements correspond to the first slice (back to front) of the 512 slices comprising the (half, top to bottom) data set.

Description—a text-based description provides details on the material being observed for each data set, including the date and hour that the sample was actually imaged at the ESRF facility.

**Fig. 2:**
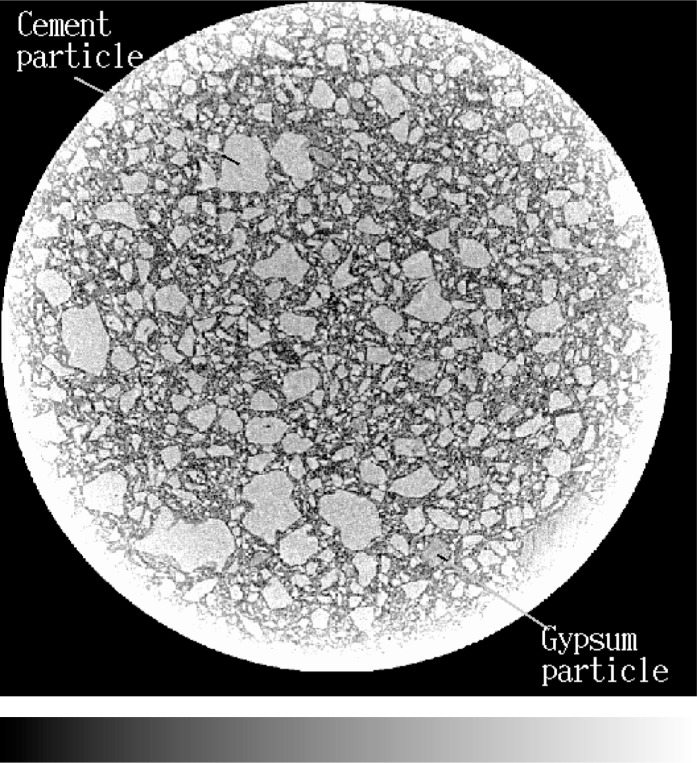
2-D image (1024 pixels by 1024 pixels) of slice 151 for unhydrated cement powder. Bright grey particles are unhydrated cement and dark grey is air-filled porosity. Greylevel bar at bottom of image indicates greylevel intensity variation from 0 (left) to 255 (right). The diameter of all slices is approximately 1 mm.

2-D slice images (JPEG format)—generally, three or more complete 1024 × 1024 2-D images are produced representing the top, middle, and bottom slices of a 300 pixel thick subsection of the 3-D microstructure (see Fig. 2 for an example). These images were used to determine the best region from which to extract the 300 voxel by 300 voxel by 300 voxel subvolumes used in the fly-through animations, generally avoiding regions that contained processing flaws or imaging artifacts.

3-D image subvolume—a 300 voxel by 300 voxel by 300 voxel subvolume was selected from each data set and stored in a 1 byte per voxel data file.

Movie files (QuickTime compatible format)—for each 300^3^ subvolume, a fly-through animation has been created. For this, each of the 300 2-D slices are viewed in sequence, giving the illusion that one is flying through the microstructure.

### 3.2 Computer Programs

The Visible Cement Data Set website also contains a variety of computer programs, written in the C programming language, to aid in the processing of three-dimensional data sets. The programs may be downloaded (via FTP), compiled, and applied to the data sets also available from the website. The following computer programs are currently available:

extract—program to extract an arbitrary subvolume of data from the 512 × 1024 × 1024 volumes;

makehist—program to produce a greylevel histogram file (named makehist.out) for a 3-D image;

segment—program to produce a segmented (phase) image from an initial greylevel image by applying a multi-level thresholding operation [17];

median and mediangrey—programs to apply a median filter [17] to either a segmented (median) 3-D image or to an initial (mediangrey) greylevel format image;

clusterid—program to apply a “burning” algorithm [18] to identify individual particle clusters in a 3-D image;

percolate—program to assess the percolation or connectivity of a “phase” in a 3-D segmented image [18];

### 3.3 Example Images, Visualizations, and Observations

#### 3.3.1 Cement Pastes

Figure 2 provides a complete 2-D (slice) image for the data set for the dry cement powder with no addition of water. A dense packing of bright cement particles surrounded by darker air space is clearly visible. In the two-dimensional image, particle sizes are observed to range between several micrometers and about 100 µm, as is typical of most portland cements [9]. There is some indication of variable levels of brightness within individual cement particles, corresponding to the different cement minerals (silicates and aluminates). Additionally, gypsum particles present in the cement powder appear darker and more uniform than the cement particles. There is some evidence of slight experimental/imaging artifacts, as evidenced by the bright ring surrounding the exterior of the sample. For this reason, a 300 voxel by 300 voxel by 300 voxel subvolume was selected from the exact center of the data set in the *xy* plane. Based on analysis of the greylevel histogram obtained for this subvolume and a subsequent segmentation of the subvolume into particles and air (porosity), the initial packing of cement particles would correspond to a *w/c* of about 0.28 if the air in the tube mold were replaced by water. Individual cement particles could be extracted from this data set and characterized with respect to size and shape, as has been performed previously for aggregate particles [7].

**Fig. 3:**
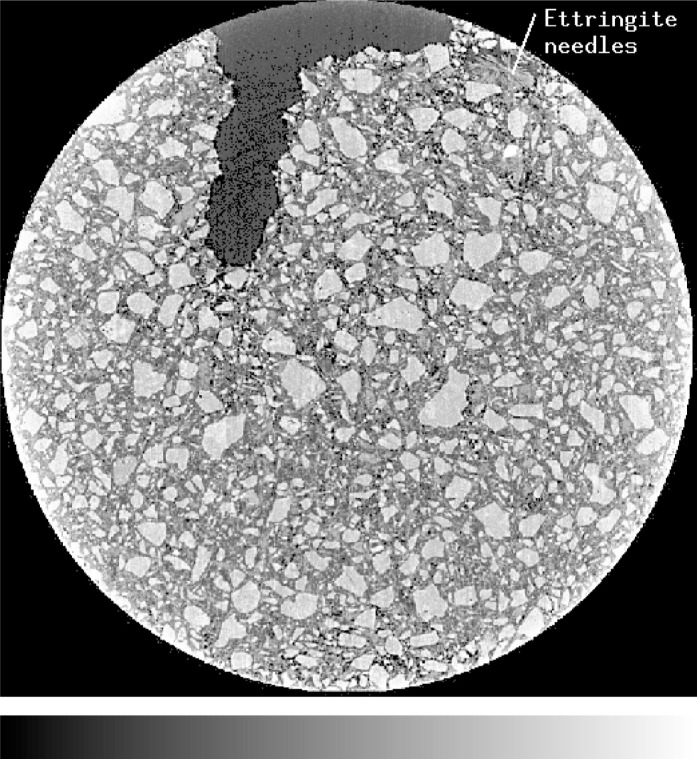
2-D image (1024 pixels by 1024 pixels) of slice 151 for *w/c* = 0.35 cement paste hydrated for 16 h. Bright grey particles are unhydrated cement, medium grey is cement hydration products, and dark grey are water-filled or air-filled pores. Some indication of ettringite needles can be seen in the upper right portion of the image. The large void region in the top central region of the image is a processing flaw due to improper compaction in the tube mold.

Figure 3 shows a 2-D slice image for the *w/c* = 0.35 cement paste hydrated for 16 h. Many unhydrated cement particles are still visible in this image. Different types of hydration products are seen to fill the initial water-filled space between the particles. Because the sample was cured under sealed conditions, some of the original water-filled pores have been converted to air (water vapor)-filled pores due to the chemical shrinkage and self-desiccation that occur during the hydration reactions [19].

**Fig. 4:**
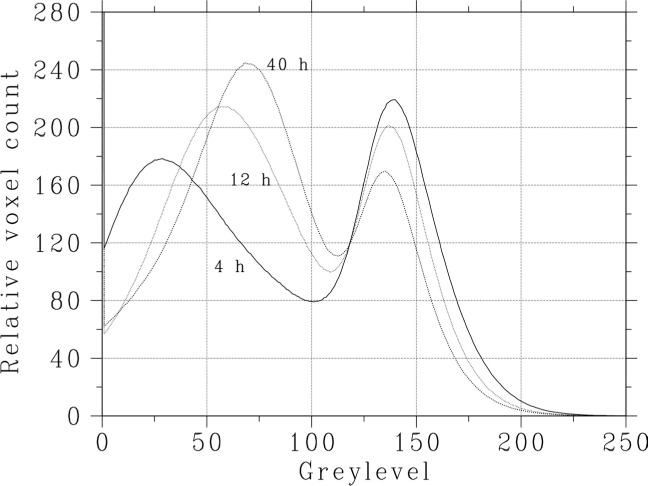
Greylevel histograms for 3-D (300 voxel by 300 voxel by 300 voxel) subvolumes of *w/c* = 0.35 cement pastes hydrated for various times.

As shown in Fig. 4, it is informative to view the greylevel histograms for the subvolumes as a function of hydration time. In Fig. 4, the disappearance of cement (high greylevels) and water (low greylevels) over time to create hydration products (middle greylevels) can be clearly distinguished. Based on these histograms, a segmentation of the subvolumes into water/air, hydration products, and unhydrated cement particles was attempted. Water and air-filled pores were selected as all voxels with a greylevel below 46. Hydration products were selected as having a greylevel between 46 and the local minimum in the range of (99, 120) shown for each curve in Fig. 4. Unhydrated cement was identified as all voxels having a greylevel greater than this local minima. Based on this segmentation, the starting *w/c* and degree of hydration of each data set could be estimated. The following estimates of *w/c* were thus obtained for 300 voxel by 300 voxel by 300 voxel portions of the top and bottom halves of each data set, respectively: 0.28 and 0.285 for the 4 h data set, 0.29 and 0.27 for the 12 h, and 0.335 and 0.317 for the 40 h. All of these are lower than the nominal *w/c* = 0.35 used to prepare the cement paste, suggesting that extrusion into the very small tube molds may have lowered the paste *w/c* by expelling water and densifying the cement pastes. It is interesting that the lowest obtained average value of 0.28 is very close to the value obtained for the data set based on a packing of dry cement particles directly into the tube. The determined degree of hydrations (volume fraction of the cement which has reacted) for the subvolumes from the top and bottom halves of each data set are then: 0.19 and 0.21 at 4 h, 0.36 and 0.36 at 12 h, and 0.47 and 0.46 at 40 h. These values can be contrasted against previously measured values (based on loss on ignition measurements) for a *w/c* = 0.3 cement paste of: 0.19 at 8 h, 0.40 at 24 h, and 0.49 at 72 h [11]. The comparison is reasonable, with some suggestion that the x rays may be accelerating the hydration at early times. To assess the possibility of an acceleration due to local heating of the specimen within the x-ray beam, a thermocouple was inserted into one of the specimens during an image acquisition. A slight temperature rise on the order of 2 °C was measured during the image acquisition, suggesting minimal acceleration due to thermal effects.

**Fig. 5:**
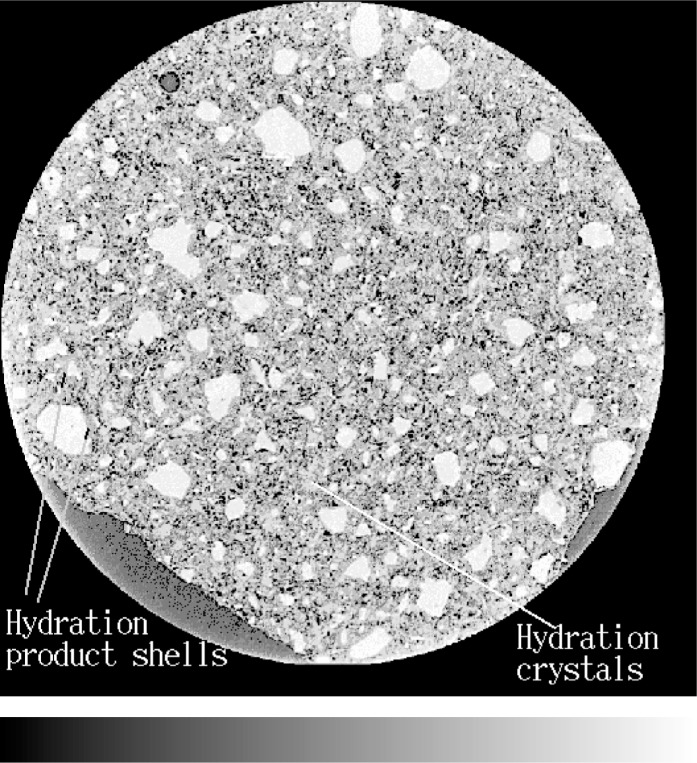
2-D image (1024 pixels by 1024 pixels) of slice 151 for *w/c* = 0.45 cement paste hydrated for 137 h. Bright grey particles are unhydrated cement, medium greys are cement hydration products, and dark grey are water-filled or air-filled pores. Fewer cement particles are present than in the previous image, due to the higher *w/c* and the longer hydration time.

**Fig. 6:**
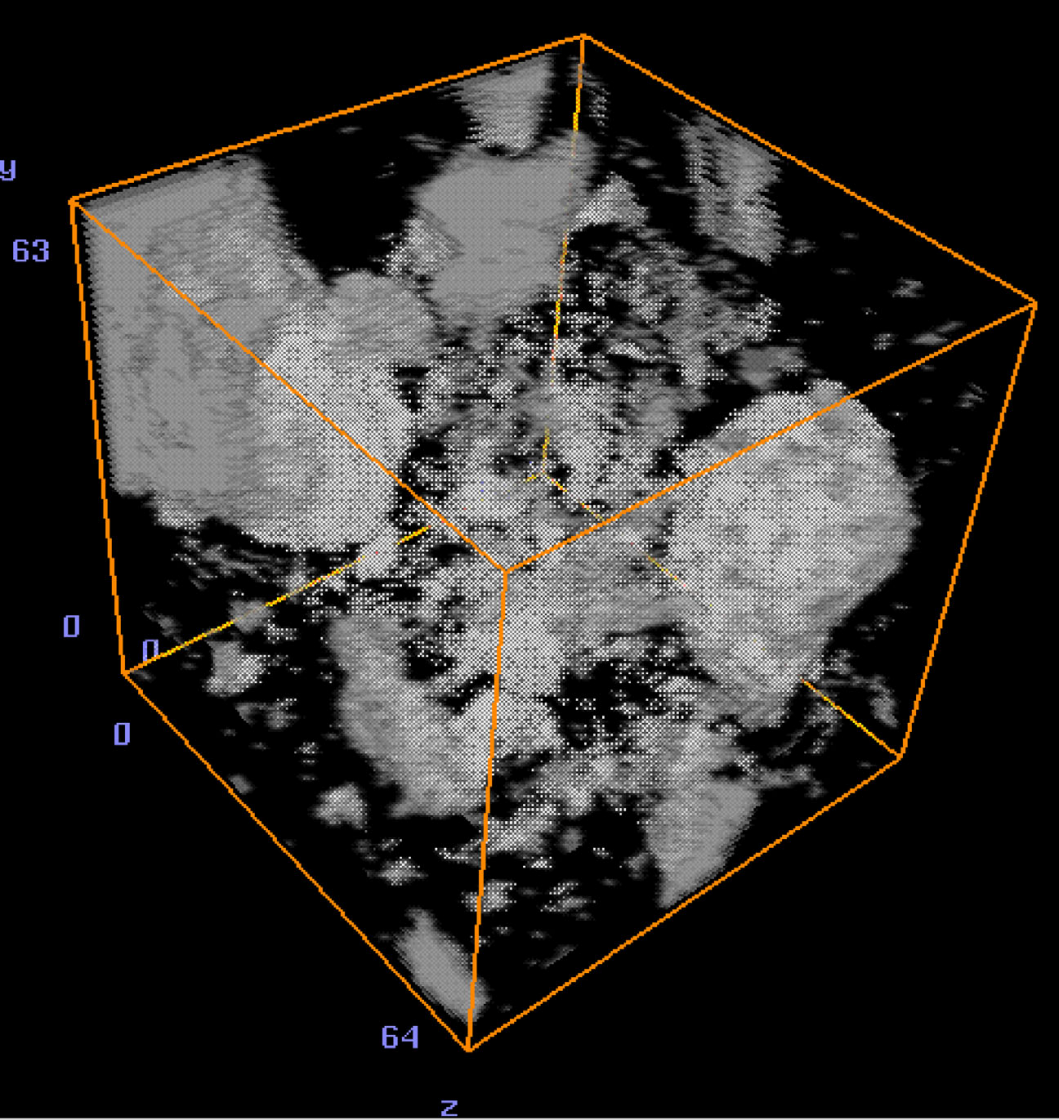
3-D image (64 voxels by 64 voxels by 64 voxels) for *w/c* = 0.45 cement paste hydrated for 137 h. Only the unhydrated cement particles are shown for clarity.

Figure 5 shows a 2-D slice for hydrated cement paste prepared at a *w/c* of 0.45 and hydrated for 137 h while Fig. 6 shows a 3-D image of a small subvolume of the same paste with only the unhydrated cement particle “cores” shown. Due to the higher *w/c* and the longer hydration time, there are fewer unhydrated cement particles in this image than in that for the *w/c* = 0.35 paste shown in Fig. 3. Upon careful examination of the image, individual needles and crystals of hydration products can be clearly observed. Shells of hydration product are visible around each of the larger unhydrated cement particles. Analyzing the greylevel histogram in a manner similar to that described above for the *w/c* = 0.35 pastes, estimates of *w/c* and the degree of hydration of 0.47 and 0.62, respectively, are obtained. This degree of hydration value compares favorably to the values previously determined by loss on ignition for a *w/c* = 0.45 paste [11]: 0.60 after 72 h and 0.70 after 168 h. Thus, the microtomography-determined values for *w/c* and degree of hydration for this data set both appear reasonable. For the 300^3^ subvolume, the segmented capillary pores (31.7 % of the overall volume) were evaluated using the program **percolate.c**. Using a burning algorithm, it was determined that about 98 % of the pores are part of a percolated (connected) pathway across the microstructure, indicating a highly connected “pore network.” In future microtomography experiments, it would be of interest to view well-hydrated (e.g., several months) specimens to investigate the de-percolation of the capillary pores that is expected to occur around 20 % porosity [20, 21].

#### 3.3.2 Plaster of Paris

**Fig. 7:**
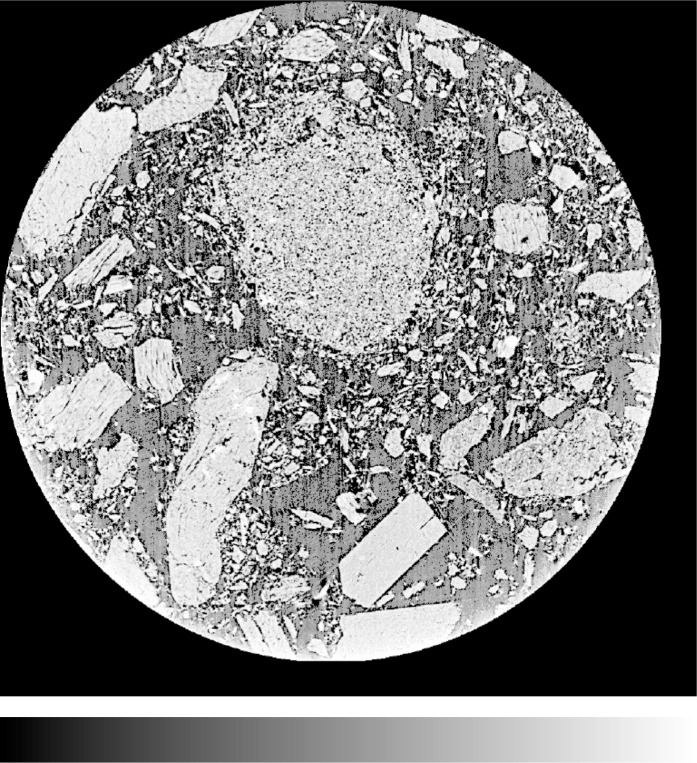
2-D image (1024 pixels by 1024 pixels) of slice 151 for dry Plaster of Paris powder.

Figure 7 shows a 2-D image for the dry Plaster of Paris powder. A wide variety of different size and shape particles are present in this material. Some of the particles have a very dense structure while others clearly exhibit some sort of microporosity and internal flaws, which could have a large influence on their reactivity with water. In this two-dimensional image, particle sizes are seen to range from a few micrometers up to about 300 µm (a somewhat wider range than that observed for the cement particles).

**Fig. 8:**
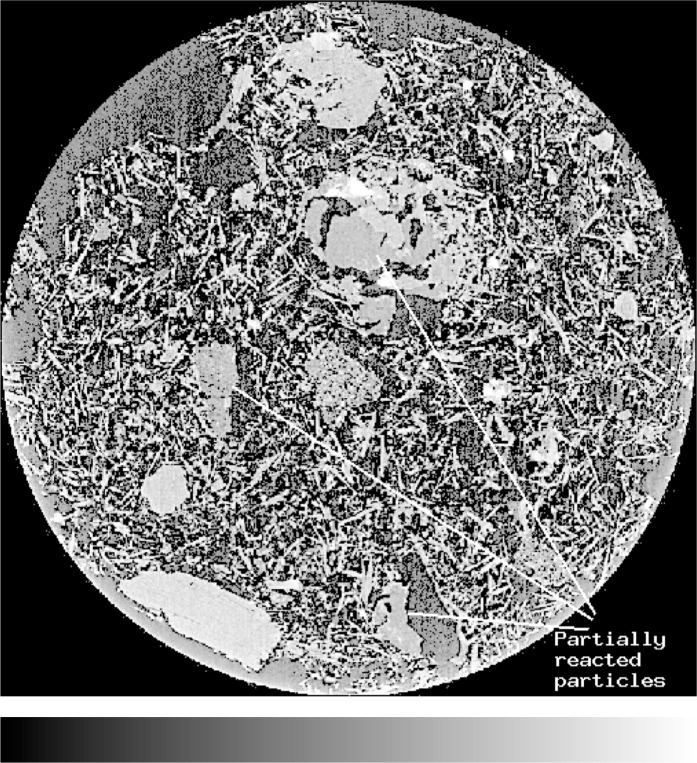
2-D image (1024 pixels by 1024 pixels) of slice 251 for *w/s* = 1.0 Plaster of Paris hydrated for 15.5 h. Large grey particles are unhydrated calcium sulfate hemihydrate. Grey needles (typically about 50 µm in length) are calcium sulfate dihydrate product. “Pore” impressions of totally and partially reacted starting particles are easily visible.

**Fig. 9:**
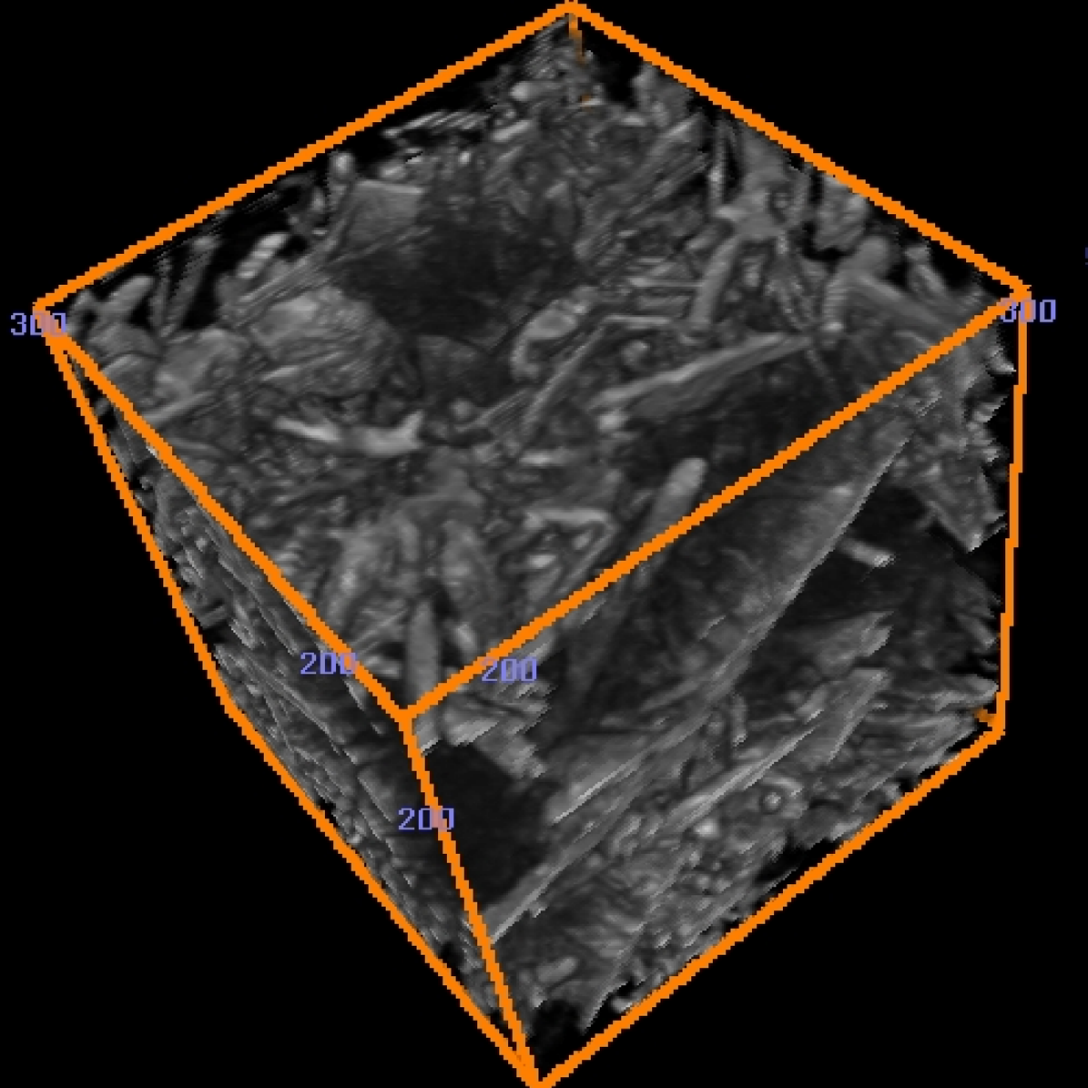
3-D image (100 voxels by 100 voxels by 100 voxels) of a portion of the *w/s* = 1.0 Plaster of Paris hydrated for 4 h. A variety of needle-like and flat plate crystals of calcium sulfate dihydrate can be seen.

Figure 8 provides a 2-D image of the Plaster of Paris microstructure after 15.5 h of hydration time while Fig. 9 provides a 3-D image of a portion of the microstructure after 4 h of hydration. These images are vastly different from that of the dry powder in Fig. 7. Numerous needles of calcium sulfate dihydrate reaction product are present in the hydrated systems. Partially reacted calcium sulfate hemihydrate particles and completely reacted particle “shells” can also be clearly observed in the 2-D image. In some cases, reaction products appear to have deposited within a portion of a partially reacted calcium sulfate hemihydrate particle. Because the morphology of calcium sulfate dihydrate crystals is strongly dependent on chemical admixtures added to the mixture and in turn has a large influence on mechanical and other physical properties, microtomography may provide a powerful tool for quantitatively evaluating the influence of these chemical admixtures on microstructure and performance. Because the elastic properties of both forms of calcium sulfate are known, these 3-D images could be used as input into a finite element software package [12] to compute the elastic properties of the material as a function of degree of hydration, *w/s*, chemical additives, etc., as has been done previously for computer model microstructures designed to mimic the Plaster of Paris system [22].

#### 3.3.3 Brick

**Fig. 10:**
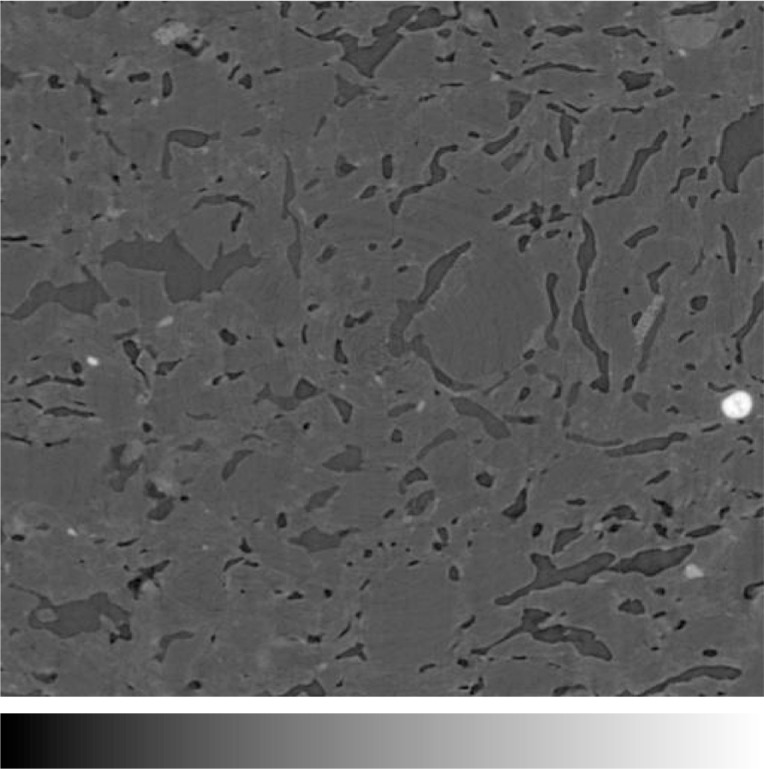
2-D image (512 pixels by 512 pixels) of the clinker brick microstructure. Slit-like pores are dark grey and solid brick material is lighter grey. The image is approximately 0.5 mm by 0.5 mm.

The clinker brick had been imaged previously at the microtomography facility in 1996, at a voxel size of 6.67 µm per pixel [6]. At this resolution, it was difficult to properly resolve many of the slit-like pores present in the material. However, as shown in Fig. 10, at a resolution of 0.95 µm, these (dark) pores are clearly visible. A simple thresholding (segmentation) could be used to isolate the porosity in the three-dimensional microstructure and examine percolation and transport properties such as conductivity [12] and permeability, as was performed previously for the microstructures obtained at the lower resolution [6]. According to physical measurements, the clinker brick has an average porosity of 20 % [6], so in this case, the threshold greylevel value used to separate solids from pores could be selected so as to closely match this porosity value in the segmented image. While the porosity appears as discontinuous isolated pores in two dimensions (Fig. 10), in three dimensions it is percolated. This serves to illustrate the importance of obtaining and analyzing three-dimensional images of a material’s microstructure, as opposed to attempting to infer three-dimensional information from two-dimensional images, in agreement with previous conclusions [22].

## 4. Summary and Prospectus

The Visible Cement Data Set website has been created and is currently being populated with the various data sets collected during the experiments conducted at the ESRF facilities. The three-dimensional data sets available from the site are presently unique in the world due to their high spatial resolution (voxel size less than one micrometer). The data sets should find usage in a variety of research and educational environments as the web site has been created with the primary objective of increasing the accessibility of these unique data sets. While a few example applications (determination of *w/c* and degree of hydration, assessment of percolation, computation of physical properties) have been discussed and presented in this paper, it is likely that other uses for this three-dimensional data will be discovered and implemented over time.
